# Angiotensin converting enzyme DD genotype is associated with acute coronary syndrome severity and sudden cardiac death in Taiwan: a case-control emergency room study

**DOI:** 10.1186/1471-2261-12-6

**Published:** 2012-02-15

**Authors:** Ying-Hsin Chen, Jui-Ming Liu, Ren-Jun Hsu, Sheng-Chuan Hu, Horng-Jyh Harn, Shee-Ping Chen, Jing-Ren Jeng, Chieh-Lin Wu, Jar-Yi Ho, Cheng-Ping Yu

**Affiliations:** 1 Institute of Medical Sciences, Tzu-Chi University, Hualien, Taiwan; 2 Department of Emergency Medicine, Tri-Service General Hospital, National Defense Medical Center, Taipei, Taiwan; 3 Division of Urology, Department of Surgery, Chang Gung Memorial Hospital and Graduate Institute of Clinical Medical Sciences, Taoyuan, Taiwan; 4 Department of Pathology, and Graduate Institute of Pathology and Parasitology, Tri-Service General Hospital, National Defense Medical Center, Taipei, Taiwan; 5 Biobank Management Center of Tri-Service General Hospital, National Defense Medical Center, Taipei, Taiwan; 6 Department of Emergency Medicine, Buddhist Tzu-Chi General Hospital, Tzu-Chi University, Hualien, Taiwan; 7 Department of Pathology, China Medical University Hospital, China Medical University, Taichung, Taiwan; 8 Tzu-Chi Stem Cell Centre, Buddhist Tzu-Chi General Hospital, Hualien, Taiwan; 9 Division of Cardiology, Buddhist Tzu-Chi General Hospital, Tzu-Chi University, Hualien, Taiwan; 10 Graduate Institutes of Life Sciences, National Defense Medical Center, Taipei, Taiwan; 11 Department of Pathology, Tri-Service General Hospital, 114 No.161, Sec. 6, Minquan E. Rd, Neihu Dist, Taipei City 114, Taiwan

**Keywords:** Acute coronary syndrome, Angiotensin converting enzyme, Genotype, Pharmacogenomics, Sudden cardiac death

## Abstract

**Background:**

Angiotensin converting enzyme (ACE) gene insertion/deletion (I/D) polymorphisms have been associated with acute coronary syndrome (ACS); however, several controversial results have also been found in different studied populations. This hospital-based, emergency room, case-control study in Taiwan retrospectively investigated 111 ACS patients, and 195 non-coronary subjects as a control group, to study the effects of ACE I/D polymorphism in the most urgent ACS patients. ACE I/D polymorphisms were determined by polymerase chain reaction-based assays and their associations with ACS risk, severity, and sudden cardiac death were determined.

**Results:**

The ACE DD genotype was associated with ACS incidence. The DD genotype was associated with a significant 4-fold higher risk of ACS in multivariate analysis (odds ratio (OR) = 4.295; 95% confidence interval (CI): 1.436-12.851, p = 0.009), and a 3.35-fold higher risk of acute myocardial infarction. DD genotype carriers also had more than 3-fold higher risks of stenosis in all the three coronary arteries, left anterior descending artery infarction, and anterior wall infarction. In addition, the DD genotype was also associated with a higher risk of sudden cardiac death (OR = 6.484, 95% CI: 1.036-40.598, p = 0.046).

**Conclusions:**

This study demonstrated that the ACE DD genotype is an independent risk factor for ACS, and in particular, for acute myocardial infarction. In addition, the ACE DD genotype is also associated with greater ACS severity and a higher risk of sudden cardiac death. ACE genotyping is recommended for patients with a history of ACS, and more intensive preventive care is suggested for patients with the DD genotype.

## Background

Acute coronary syndrome (ACS) is a common disease in the elderly, and a major determinant of morbidity and mortality in all races, ethnicities, and cultures [1]. The epidemic of coronary artery disease (CAD), especially its manifestation as ACS, is a global issue that accounts for more than 80% of the burden of this disease in developing countries [2], and results in approximately 30% of all deaths worldwide each year [3]. The spectrum of ACS disease severity ranges from unstable angina pectoris (UAP) to acute myocardial infarction (AMI), including ST-segment elevation myocardial infarction (STEMI) and non-STEMI (NSTEMI). Sudden cardiac arrest is a major event of heart failure, and sudden cardiac death frequently presents as the first and only manifestation of previously unrecognized ischemic heart disease [4]. The prognosis of AMI-related heart failure depends on the extent and severity of ventricular dysfunction and morbidity [5]. Clinically, heart failure usually results in a 20-25% reduction in left ventricular contractility, and a reduction of more than 40% leads to cardiogenic shock [6].

Angiotensin converting enzyme (ACE) is a membranous zinc-containing decarboxy-peptidase that catalyzes the conversion of angiotensin I to angiotensin II and the inactivation of bradykinin via the kallikrein-kininogen system [6]. The insertion (I)/deletion (D) polymorphism in intron 16 of the ACE gene is designated as the presence or absence of a 287 bp Alu repeat [7,8]. The D allele is associated with high serum ACE in the order homozygote deletion (DD) > heterozygote (ID) > homozygote insertion (II) [7,8]. Cardiac ACE activity is also higher in DD homozygote carriers [9]. Treatment with ACE inhibitors after MI has been found to attenuate pathologic left ventricle remodeling, the incidence of heart failure, and mortality [10,11]. The ACE DD genotype or D allele has been associated with MI by increasing the risk of left ventricular enlargement or remodeling [12] and ischemic or idiopathic dilated cardiomyopathy [13,14], though there are also several reports which found no association [15-17]. Genetic polymorphisms in ACE-related pathways, including the angiotensin II type 1 receptor (AT1R) and AT2R, have been associated with MI-related arrhythmias and risk of sudden cardiac arrest [18]. ACE DD and AT1R 1166 CC genotypes have been linked with a greater risk of diastolic heart failure [19].

The DD genotype or D allele has been most associated with increased risk of ACS, ACS severity and cardiovascular death. However, inconsistencies in the clinical significance in previous reports suggested that the influence of the ACE I/D genotypes may differ across the ACS spectrum. For this reason, we carried out a retrospective emergency room-based case-control study to evaluate the association of the ACE I/D polymorphism with the most urgent ACS events. Therefore, this study addressed ACE genotypes and ACS incidence, severity, and sudden cardiac death within the recruited population.

## Methods

### Subjects

This retrospective case-control study included 111 acute coronary syndrome (ACS) patients and 195 non-coronary subjects (NCS) who were treated in the emergency room of Tri-Service General Hospital (TSGH) between July 2005 and June 2010. The subjects in this study were all Taiwanese and 20 years of age or older. The study was approved by the institutional review board. Clinical and follow-up information were obtained from the patients' medical records, and peripheral blood samples were obtained from the Biobank of TSGH. Peripheral blood samples of each patient were collected by the Biobank during the follow-up with informed consent; two of the blood samples of each patient were used to confirm the ACE genotype. Among the ACS patients, 91 were identified with ACS by coronary angiography (defined by > 75% stenosis in any major coronary artery), while 20 ACS patients did not receive coronary angiography because of their condition [20]. Fifty four ACS patients experienced STEMI, with cardiac chest pain, serologic evidence of myonecrosis, and persistent (> 20 min) ST-segment elevation. The other 57 ACS patients had either UAP or NSTEMI, that was clinically documented as: (1) UAP when cardiac chest pain was new or worsening without serologic evidence of myonecrosis (i.e., no elevation of serum troponin or creatine kinase MB isoenzyme concentration), or dynamic eletrocardiographic (ECG) changes (i.e., ST depression and/or T wave inversion); (2) NSTEMI when the patient had cardiac chest pain and serologic evidence of myocardial necrosis in the absence of ST-segment elevation on the ECG [21]. The NCS had no clinical evidence of CAD including: (1) no history of typical angina pectoris; (2) no abnormal Q wave or ST-T changes in the resting electrocardiogram; and (3) a negative Master exercise test. The NCS constituted the control group and included 135 men and 60 women. The presence of hypertension, diabetes mellitus, hypercholesterolemia or smoking was obtained from the patients' charts or from the records of examination during follow-up medication or hospitalization. In this study, hypercholesterolemia was defined as a serum total cholesterol level of 200 mg/dl or more. Diagnosis of hypertension and diabetes mellitus were performed according to World Health Organization criteria. A smoking habit was defined as a daily intake of > 10 cigarettes. Sudden cardiac death was defined as death within 24 hours of registration in the emergency room, and ACS was recorded as the cause of death after failure of standard cardiac arrest treatment. Other risk factors were stratified as indicated in each table.

### Polymerase chain reaction (PCR) for detection of ACE Insert/Delete (I/D) polymorphism

Genomic DNA was extracted from peripheral blood using the Qiagen PureGene DNA Purification Kit according to the manufacturer's protocol. The presence of the I and D alleles of the ACE gene was detected according to the method in Tiret et al [8]. The sequence of the sense primer was 5'-CTGGAGACCACTCCCATCCTTTCT-3', and the antisense primer was 5'-GATGTGGCCATCACATTCGTCAGAT-3'. PCR was carried out with 80 ng genomic DNA in a final volume of 50 μl containing 3 mmol/l magnesium chloride, 50 mmol/l potassium chloride, 10 mmol/l Tris-HCl (pH8.4), 0.1 mg/dl gelatin, 10 pmol of each primer, 200 μmol of each dNTP and 1 unit Taq-DNA polymerase (Promega, Madison, WI, USA). Amplification was performed in a DNA thermal cycler 480 (Perkin-Elmer, Norwalk, CT, USA) involving 1 minute of denaturation at 94°C, 1 minute of annealing at 55°C and 2 minutes of extension at 72°C for 35 cycles. In the last cycle, the extension step was carried out for 10 minutes. Thereafter, 10 μl of amplified product was electrophoresed in a 2% agarose gel containing 0.5 μg/ml of ethidium bromide and was subsequently visually inspected under ultraviolet light for appropriately sized bands of DNA. The I allele manifested as a 490 bp band, and the D allele was seen as a 190 bp band of DNA. Besides, all DD genotype samples were confirmed using the primers: forward, 5'-TCGGACCACAGCGCCCGCCACTAC-3'; and reverse, 5'-TCGCCAGCCCTCCCATGCCCATAA-3' with identical PCR conditions except for an annealing temperature of 67°C, that produced a 335-bp product only in the presence of the I allele [22].

### Data analysis

Patient age at diagnosis was recorded as a continuous variable and shown as mean ± standard deviation. The odds ratios (ORs) and corresponding 95% confidence intervals (CIs) for assessing the effect of the ACE I/D genotype distribution and allele frequencies on ACS were calculated by logistic regression analysis with adjustment for relevant significant variables. All the statistical analyses were performed using SPSS 16.0 and Excel 2007. For all statistical tests, p values were two-sided and the level of significance was set at 0.05.

## Results

### Risk factors for ACS

Comparisons of the distributions of demographic and relevant clinical risk factors between ACS patients and NCS controls and the estimated OR for each risk factor are listed in Table 1. Patients diagnosed as ACS were significantly older than control NCS (mean age 64.99 ± 13.85 years and 42.44 ± 11.91 years, respectively). ACS patients were also more likely to have higher systolic or diastolic blood pressure and diabetes mellitus than NCS. The groups were similar with respect to gender and body mass index. In the univariate logistic regression model, older age, higher systolic blood pressure or diastolic blood pressure and the presence of diabetes mellitus were found to be significant risk factors for ACS.

**Table 1:** Univariate and multivariate analyses of ACS risk factors

Subject characteristics	ACS (n = 111)	NCS (n = 195)	Univariate		Multivariate	
			
			OR (95%CI)	p*	OR (95% CI)	P*
ACE-genotypes						

II	37	78	1 (ref.)		1 (ref.)	

ID	51	99	1.086 (0.648-1.821)	0.754	1.284 (0.595-2.773)	0.524

DD	23	18	**2.694 (1.298**-**5.592)**	**0.008**	**4.295 (1.436**-**12.851)**	**0.009**

Demographic factors						

Age	64.99 ± 13.85	42.44 ± 11.91	**1.127 (1.099**-**1.156)**	**< 0.001**	**1.114 (1.082**-**1.147)**	**< 0.001**

Sex -Female	25	60	1 (ref.)			

Male	86	135	1.529 (0.892-2.622)	0.123	-	-

Clinical risk factors						

BMI (kg/m^2^) < 27	99	174	1.004 (0.474-2.128)	0.991	-	-

≥ 27	12	21				

Systolic blood pressure	73	181	1 (ref.)		1 (ref.)	

< 140 mmHg						

≥ 140 mmHg	38	14	**6.730 (3.443**-**13.156)**	**< 0.001**	1.765 (0.525-5.934)	0.358

Diastolic blood pressure	88	179	1 (ref.)		1 (ref.)	

< 90 mmHg						

≥ 90 mmHg	23	16	**2.924 (1.471**-**5.813)**	**0.002**	1.425 (0.497-4.089)	0.510

Diabetes Mellitus - No	75	187	1 (ref.)		1 (ref.)	

Yes	36	8	**11.220 (4.983**-**25.261)**	**< 0.001**	2.396 (0.869-6.610)	0.091

On the basis of logistic regression, with statistical significance (p < 0.05) shown in bold *ACS*: acute coronary syndrome, *NCS*: non-coronary subjects, *BMI*: body mass index

### ACE DD genotype as an independent risk factor for ACS

Table 1 illustrates that ACS patients demonstrated a lower proportion of II genotypes (33.33%) and ID genotypes (45.95%) but higher proportions of DD genotypes (20.72%) than the control population (II, 40.00%; ID, 50.77%; DD, 9.23%). The ACE DD genotype conferred a 2.69-fold higher risk of ACS compared with the II genotype (95% CI: 1.298-5.592, p = 0.008, logistic regression). After controlling for other risk factors, the DD genotype was still significantly associated with ACS, conferring a 4-fold higher risk (OR = 4.295; 95% CI: 1.436-12.851). Age was the only other independent risk factor under multivariate logistic regression, which was in accordance with other studies [1,23].

### ACE DD genotype associated with ACS type, severity and mortality

To further evaluate the etiologic effects of ACE I/D polymorphisms in ACS, we analyzed the association between ACE genotypes and different ACS manifestations (UAP and AMI), complications (hypertension, diabetes mellitus, hypercholesterolemia), relevant clinical risk factors (systolic blood pressure, diastolic blood pressure, total blood cholesterol and triglyceride and body mass index), clinical history (heart failure, CAD, MI and family history) and history of smoking. As shown in Table 2, the DD genotype conferred a significant 3.35-fold higher risk of AMI (Table 2, 95% CI: 1.111-10.115, p = 0.032, logistic regression), however there was no significant association between ACE I/D polymorphisms and any other risk factor among the recruited ACS group. A multivariate analysis was not performed as only ACS was significantly associated with ACE I/D polymorphisms. The foregoing results also supported previous studies that the ACE D allele was associated with higher ACE activity [7-9], converting more angiotensin II and thus stimulating inflammatory/fibrogenic responses and scar formation during myocardial infarction, or inducing reactive oxygen species to cause myocardial damage in the border zones and enlargement of the infarct size [24].

**Table 2:** Association between ACE I/D polymorphisms and ACS risk factors

ACE genotypes	II (n = 37)		ID (n = 51)			DD (n = 23)		
	
ACS risk factors				Univariate			Univariate	
	
	n		n	OR (95%CI)	p*	n	OR (95%CI)	p*
ACS spectrum								

UAP (n = 57)	22	1 (ref.)	28			7		

AMI (n = 54)	15		23	1.205 (0.511-2.839)	0.670	16	**3.352 (1.111**-**10.115)**	**0.032**

Demographic factors								

Age (mean)	67.7 ± 11.1	1 (ref.)	64.2 ± 13.5	0.981 (0.949-1.013)	0.232	62.4 ± 18.1	0.972 (0.935-1.010)	0.149

Sex -female	10	1 (ref.)	8			7		

male	27		43	2.352 (0.733-7.543)	0.150	16	1.181 (0.375-3.719)	0.776

Complications								

Hypertension- No	10	1 (ref.)	20			11		

- Yes	27		31	0.574 (0.229-1.437)	0.236	12	0.404 (0.135-1.206)	0.104

Diabetes Mellitus-No	25	1 (ref.)	33			17		

- Yes	12		18	0.880 (0.359-2.157)	0.780	6	1.360 (0.427-4.328)	0.603

Hypercholesterolemia-No	31	1 (ref.)	39			19		

- Yes	6		12	0.629 (0.212-1.866)	0.403	4	0.919 (0.229-3.684)	0.905

Other clinical risk factors								

Systolic blood pressure	22	1 (ref.)	38			13		

< 140 mmHg								

≥ 140 mmHg	15		13	0.502 (0.202-1.246)	0.137	10	1.128 (0.393-3.236)	0.822

Diastolic blood pressure	27	1 (ref.)	45			16		

< 90 mmHg								

≥ 90 mmHg	10		6	0.360 (0.118-1.102)	0.074	7	1.181 (0.375-3.719)	0.776

Total cholesterol < 200 mg/dl	31	1 (ref.)	46			17		

≥ 200 mg/dl	6		5	0.562 (0.158-2.002)	0.374	6	1.824 (0.509-6.538)	0.356

Triglyceride < 150 mg/dl	34	1(ref.)	42			17		

≥ 150 mg/dl	3		9	1.768 (0.500-6.251)	0.377	6	2.912 (0.722-11.736)	0.133

BMI (kg/m^2^) < 27	35	1 (ref.)	43			21		

≥ 27	2		8	3.256 (0.649-16.328)	0.151	2	1.667 (0.218-12.732)	0.622

History								

Heart failure history-No	29	1 (ref.)	41			16		

-Yes	8		10	0.884 (0.311-2.512)	0.817	7	1.586 (0.485-5.181)	0.445

CAD history -No	22	1 (ref.)	32			17		

-Yes	15		19	0.871 (0.366-2.074)	0.755	6	0.518 (0.166-1.617)	0.257

MI history -No	34	1 (ref.)	46			21		

-Yes	3		5	1.232 (0.275-5.512)	0.785	2	1.079 (0.166-7.004)	0.936

Family history of ACS	35	1 (ref.)	50			22		

-Yes	2		1	0.350 (0.031-4.012)	0.399	1	0.795 (0.068-9.301)	0.855

Smoking	20	1 (ref.)	29			11		

-Yes	17		22	0.892 (0.381-2.091)	0.793	12	1.283 (0.452-3.641)	0.639

On the basis of logistic regression, with statistical significance (p < 0.05) shown in bold. Multivariate analyses were not performed as only the DD genotype was significantly associated with AMI

*ACE*, angiotensin converting enzyme, *UAP*: unstable angina pectoris; *AMI*: acute myocardial infarction, *CAD*: coronary artery disease; *MI*: myocardial infarction.

Subsequent analyses evaluated the possible influence of ACE I/D polymorphisms on final ACS clinical outcome, and the association between ACE I/D polymorphisms and ACS severity and mortality was determined. Interestingly, ACS patients with the DD genotype had a significantly higher risk of presenting with a greater number of stenosed vessels, with a 3.87-fold increased risk of stenosis in all three coronary arteries (Table 3, 95% CI: 1.085-13.812, p = 0.037, logistic regression). In addition, the DD genotype was also associated with a 3.08-fold higher risk of left anterior descending artery infarction (Table 3, 95% CI: 1.041-9.118, p = 0.042), and a 3.07-fold higher risk of anterior wall infarction (Table 3, 95% CI: 1.039-9.091, p = 0.043). Previous epidemiological data showed that an anterior wall myocardial infarction is usually caused by an occlusion of the left anterior descending coronary artery [25,26], and the DD genotype is highly associated with those ACS symptoms. We also found that the DD genotype was independently associated with sudden cardiac death (Table 4, OR = 6.484, 95% CI: 1.036-40.598, p = 0.046, logistic regression). This effect seemed more obvious in AMI patients (sudden cardiac death in AMI patients: genotype II = 2, ID = 2, DD = 5; and in UAP patients: genotype II = 0, ID = 2, DD = 1), but there was no statistical significance between ACE I/D polymorphism and sudden cardiac death across the ACS spectrum. A history of heart failure also conferred a 9.69-fold increased risk of sudden cardiac death which was similar to that found in previous epidemiological studies [4,5].

**Table 3:** Association between ACE I/D polymorphisms and ACS clinical severity

ACE genotypes	II (n = 37)		ID (n = 51)			DD (n = 23)		
	
Clinical symptoms				Univariate			Univariate	
	
	n		n	OR (95%CI)	p*	n	OR (95%CI)	p*
Stenosis numbers								

0 or 1 vessel	19	1 (ref.)	20			6		

2 vessels	9		12	1.267 (0.435-3.686)	0.665	6	2.006 (0.729-5.515)	0.178

3 vessels	9		19	2.111 (0.530-8.407)	0.289	11	**3.870 (1.085**-**13.812)**	**0.037**

Infarcted artery (yes vs. no)								

left anterior descending -No	23	1(ref)	22			8		

-Yes	14		29	2.166 (0.912-5.144)	0.080	15	**3.080 (1.041**-**9.118)**	**0.042**

left circumflex -No	21	1(ref)	24			14		

-Yes	16		27	1.477 (0.630-3.460)	0.370	9	0.844 (0.292-2.436)	0.753

right coronary -No	17	1(ref)	21			12		

-Yes	20		29	1.174 (0.498-2.764)	0.714	11	0.779 (0.275-2.211)	0.639

Infarction locus (yes vs. no)								

anterior wall -No	26	1(ref)	39			10		

-Yes	11		12	0.727 (0.279-1.893)	0.514	13	**3.073 (1.039**-**9.091)**	**0.043**

inferior wall -No	34	1(ref)	42			19		

-Yes	3		9	2.429 (0.609-9.679)	0.208	4	2.386 (0.482-11.803)	0.286

right ventricle myocardial -No	36	1(ref)	48			22		

-Yes	1		3	2.250 (0.225-22.533)	0.490	1	1.636 (0.097-27.511)	0.732

others -No	35	1(ref)	50			22		

-Yes	2		1	0.350 (0.031-4.012)	0.399	1	0.795 (0.068-9.301)	0.855

* On the basis of logistic regression, with statistical significance (p < 0.05) shown in bold.

**Table 4:** Association between ACE I/D polymorphisms and ACS sudden cardiac death

Subject characteristics	Survival	Expired	Univariate		Multivariate	
			
	(n = 99)	(n = 12)	OR (95%CI)	p*	OR (95% CI)	p*
ACE-genotypes						

II	35	2	1 (ref.)		1 (ref.)	

ID	47	4	1.489 (0.258-8.595)	0.656	1.659 (0.264-10.427)	0.589

DD	17	6	**6.176 (1.126**-**33.876)**	**0.036**	**6.484 (1.036**-**40.598)**	**0.046**

ACS spectrum						

UAP (n = 57)	54	3	1 (ref.)			

AMI (n = 54)	45	9	3.600 (0.919-14.100)	0.066		

Demographic factors						

Age	65.02 ± 13.40	64.85 ± 17.90	0.999 (0.956-1.043)	0.949	-	-

Sex -Female	21	4	1 (ref.)			

Male	78	8	1.857 (0.510-6.769)	0.348	-	-

Complications						

Hypertension- No	36	5	1 (ref.)		-	-

- Yes	63	7	0.800 (0.237-2.706)	0.720		

Diabetes Mellitus-No	68	7	1 (ref.)		-	-

- Yes	31	5	1.567 (0.461-5.327)	0.472		

Hypercholesterolemia-No	78	11			-	-

- Yes	21	1	0.338 (0.041-2.766)	0.312		

Other clinical risk factors						

Systolic blood pressure	67	6	1(ref.)		-	-

< 140 mmHg						

≥ 140 mmHg	32	6	2.094 (0.626-7.003)	0.230		

Diastolic blood pressure	78	10	1 (ref.)		-	-

< 90 mmHg						

≥ 90 mmHg	21	2	0.743 (0.151-3.653)	0.715		

Total cholesterol	83	11	1 (ref.)	-	-	

< 200 mg/dl						

≥ 200 mg/dl	16	1	0.472 (0.057-3.913)	0.486		

Triglyceride < 150 mg/dl	81	11	1 (ref.)	-	-	

≥ 150 mg/dl	18	1	0.409 (0.050-3.374)	0.406		

BMI (kg/m^2^) < 27	88	11	1 (ref.)	-	-	

≥ 27	11	1	0.727 (0.085-6.187)	0.771		

History						

Heart failure history-No	82	4	1 (ref.)			

-Yes	17	8	**9.647 (2.606**-**35.716)**	**0.001**	**9.698 (2.468**-**38.106)**	**0.001**

CAD history -No	63	8	1 (ref.)	-	-	

-Yes	36	4	0.875 (0.246-3.110)	0.836		

MI history -No	91	10	1 (ref.)	-	-	

-Yes	8	2	2.275 (0.423-12.224)	0.338		

Family history of ACS	96	11	1 (ref.)	-	-	

-Yes	3	1	2.909 (0.278-30.430)	0.373		

Smoking	53	7	1 (ref.)	-	-	

-Yes	46	5	0.823 (0.245-2.770)	0.753		

*On the basis of logistic regression, with statistical significance (p < 0.05) shown in bold.

## Discussion

The renin-angiotensin system (RAS) has been recognized for many years as a critical pathway for blood pressure control and kidney function [27]. This 5-year period emergency room-based study clearly demonstrates that the ACE DD genotype acts as an independent risk factor for ACS requiring emergency treatment (Table 1), and in particular for AMI (Table 2). In addition, the ACE DD genotype is also associated with greater ACS severity, including more stenosed vessels, greater occlusion of the left anterior descending branch, and a greater risk of anterior wall myocardial infarction (Table 3) and also of sudden cardiac death (Table 4).

The ACE DD genotype was reported to be associated with CAD risk in several studies including ECTIM [7], INSERM [8], ISIS [10] and CORGENE [28] cohorts, and subsequent studies revealed that the D allele or DD genotype may confer effects mainly on the onset of ACS or MI [29] by modifying ACE abundance or activity, and contributing to increases in plaque instability, ulceration and thrombosis [30]. The ACE D allele was correlated with an increase in serum or cardiac ACE, thus increasing the conversion of angiotensin I to the peptide precursor angiotensin II which induces aldosterone production [7-9]. Elevation of circulating aldosterone increases the risk of arterial hypertension and cardiac fibrosis thus inducing diastolic dysfunction, ventricular remodeling and even atherosclerosis [31]. Similar results were also found in this case-control study, with DD genotype carriers having about a four-fold higher risk of ACS than ID or II genotype carriers. In addition, age of diagnosis, systolic blood pressure, diastolic blood pressure and diabetes mellitus were also identified as ACS risk factors in univariate analyses, though only the ACE DD genotype and age of diagnosis were found to be independent risk factors. Older age is a well-documented risk factor for CAD or its manifestation as ACS [1,23], and the ACE DD genotype or D allele had been found in most studies to be a risk factor for CAD or ACS [12,28,29]. Within the ACS groups, we also compared disease type, complications, clinical history, and other relevant clinical risk factors. Interestingly, only the ACE DD genotype conferred about 3-fold risk for AMI compared with UAP and this result was also similar to most studies in different population or study cohorts [32], although some studies found no association [14,25]. There was no significance between any other analyzed risk factor and ACE I/D polymorphisms; even though DD genotype had been reported to be associated with hypertension [33] and/or diabetes mellitus [34]. Patients with the ACE DD genotype appeared to have earlier onset of ACS, with a younger mean age than ID or II carriers, but there was no statistical significance among those groups (analysis of variance p = 0.307).

Although ACE I/D polymorphisms have been comprehensively studied in CAD or ACS, there are only a few studies addressing the clinical significance of ACE genotypes on the severity of coronary lesions. In this emergency room-based, case-control study, we enrolled the most urgent ACS cases who required immediate treatment, and we found that DD genotype carriers were likely to have the most severe ACS, including stenosis in all three coronary arteries, left anterior descending artery infarction and also anterior wall infarction (Table 3). Similarly, a previous study found that DD genotype carriers had a greater number of stenosed arteries and/or dangerous infarction loci [26].

Finally, we focused this study on the effects of ACE I/D polymorphisms on sudden cardiac death within 24 hours of the ACS patients being registered in the emergency room. ACE DD genotype carriers also had a higher mortality risk than ID or II carriers. Genetic polymorphisms in ACE-related genes have been associated with risk of MI-related sudden

cardiac arrest [18], and the ACE DD genotype was found to confer an increased risk of diastolic heart failure [19,33], ACS recurrence and cardiovascular mortality [19], although controversial and negative [12,17] results have also been reported.

Polymorphisms of ACE and its related genes have also been highly associated with the efficiency of ACE inhibitor treatment and the role of ACE I/D polymorphisms are particularly well documented [35,36].

## Conclusions

In summary, this case-control study has demonstrated that the ACE DD genotype is an independent risk factor for ACS incidence, and especially for AMI. In addition, the ACE DD genotype is associated with greater ACS severity and greater risk of sudden cardiac death. This emergency room experience of patients requiring urgent ACS treatment suggests that ACE DD genotype carriers should be followed-up and treated especially carefully because of the higher relative risk of more severe ACS and sudden cardiac death. Further studies are necessary to evaluate the personal pharmacogenomic and pharmacogenetic effects of ACE I/D polymorphisms to improve ACS therapeutic strategies and prognosis.

## Competing interests

The authors declare that they have no competing interests.

## Authors' contributions

YHC, JML and RJH designed the research plan and study. YHC, JYH, RJH, SCH and CLW carried out all experiments. HJH and YHC provided clinical specimens. JYH, RJH and CPY drafted and edited the manuscript. All authors read and approved the final manuscript.

## Pre-publication history

The pre-publication history for this paper can be accessed here:

http://www.biomedcentral.com/1471-2261/12/6/prepub
